# Binding motif for RIC-3 chaperon protein in serotonin type 3A receptors

**DOI:** 10.1085/jgp.202213305

**Published:** 2023-04-07

**Authors:** Hoa Quynh Do, Michaela Jansen

**Affiliations:** 1 Department of Cell Physiology and Molecular Biophysics and Center for Membrane Protein Research, School of Medicine, Texas Tech University Health Sciences Center, Lubbock, TX, USA

## Abstract

Serotonin or 5-hydroxytryptamine type 3 (5-HT_3_) receptors belong to the family of pentameric ligand-gated ion channels (pLGICs) that are therapeutic targets for psychiatric disorders and neurological diseases. Due to structural conservation and significant sequence similarities of pLGICs’ extracellular and transmembrane domains, clinical trials for drug candidates targeting these two domains have been hampered by off-subunit modulation. With the present study, we explore the interaction interface of the 5-HT_3A_ subunit intracellular domain (ICD) with the resistance to inhibitors of choline esterase (RIC-3) protein. Previously, we have shown that RIC-3 interacts with the L1-MX segment of the ICD fused to maltose-binding protein. In the present study, synthetic L1-MX-based peptides and Ala-scanning identify positions W347, R349, and L353 as critical for binding to RIC-3. Complementary studies using full-length 5-HT_3A_ subunits confirm that the identified Ala substitutions reduce the RIC-3-mediated modulation of functional surface expression. Additionally, we find and characterize a duplication of the binding motif, DWLR…VLDR, present in both the MX-helix and the transition between the ICD MA-helix and transmembrane segment M4. Analogous Ala substitutions at W447, R449, and L454 disrupt MAM4-peptide RIC-3 interactions and reduce modulation of functional surface expression. In summary, we identify the binding motif for RIC-3 in 5-HT_3A_ subunits at two locations in the ICD, one in the MX-helix and one at the MAM4-helix transition.

## Teaser

This work identifies a binding motif in a serotonin channel (5-HT_3A_) for RIC-3 protein. The discovery positions the interaction as a potential drug target in treating 5-HT_3A_-related disorders and opens new research directions to understand channel biogenesis.

## Introduction

Serotonin or 5-hydroxytryptamine type 3 (5-HT_3_) receptors are members of the family of pentameric ligand-gated ion channels (pLGICs) that also includes nicotinic acetylcholine receptors (nAChR; Fakhfouri et al., 2019; Gibbs and Chakrapani, 2021). Gating of 5-HT_3_ channels by the endogenous agonist serotonin results in permeation by cations and signal transduction in the nervous system (Derkach et al., 1989; Reiser et al., 1989; Sugita et al., 1992; Nichols and Mollard, 1996; McMahon and Kauer, 1997; Rondé and Nichols, 1998; Basak et al., 2018a; Polovinkin et al., 2018). pLGICs’ involvement in brain–gut signaling circuitry and other non-serotonergic synaptic activities render these receptors effective and potential therapeutic targets for the treatment of conditions such as irritable bowel syndrome, chemotherapy-induced vomiting, inflammation, and psychiatric disorders (Humphrey et al., 1999; Rubenstein, 2004; Herrstedt and Dombernowsky, 2007; Spiller, 2011; Rojas and Slusher, 2012; Kurhe et al., 2014; Wu et al., 2015; Fakhfouri et al., 2019).

**Figure 1.**
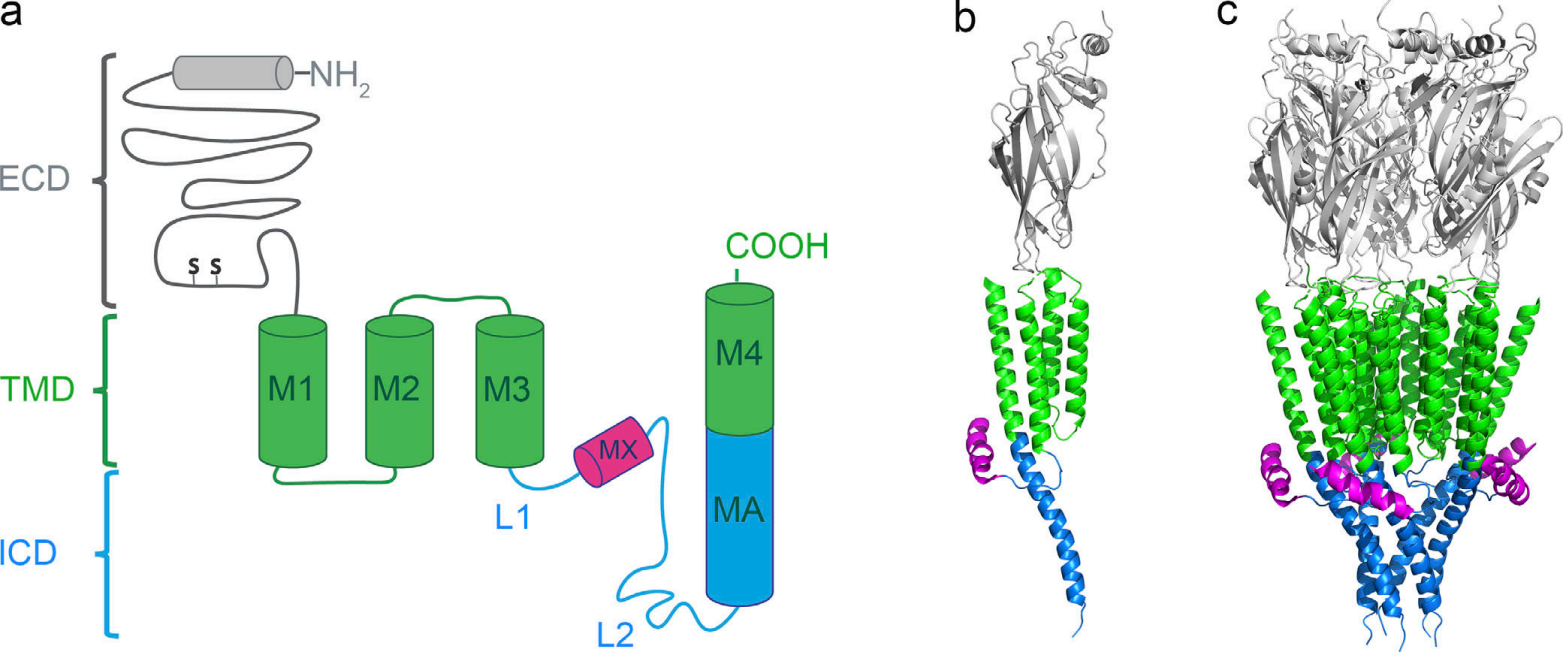
**Domain structure of the 5-HT**_**3A**_ **receptor. (a)** Schematic of a single subunit illustrating the organization of the three domains, extracellular (gray), transmembrane (green) with four α-helical segments (M1 through M4), and intracellular (blue and magenta) with a 17 amino acid long MX-helix and a MA helix of 31 amino acids in length continuous with the subsequent M4 transmembrane helix. **(b and c)** Ribbon representation of a single subunit and pentameric receptor (PDB ID 4PIR; Hassaine et al., 2014). Colors as in a. All views parallel to the membrane plane.

5-HT_3_ receptors assemble either into homopentamers from 5-HT_3A_ subunits or heteropentamers by combining 5-HT_3A_ subunits with other subunits (B through E; Davies et al., 1999; Dubin et al., 1999; Lummis, 2012). 5-HT_3A_ subunits share their molecular structure with the pLGIC family. Each homologous subunit comprises three domains: an extracellular N-terminal domain (ECD) housing agonist-binding sites, a transmembrane domain (TMD) enabling cations to cross the plasma membrane, and an intracellular domain (ICD) playing critical roles in channel conductance and functional expression (Thompson et al., 2010; Hassaine et al., 2014; Basak et al., 2018b). The transmembrane domain consists of four α-helical transmembrane-spanning segments called M1, M2, M3, and M4 (Fig. 1 a). In 5-HT_3A_ subunits, M3 and M4 are connected by a 115-amino acid segment that contributes the main part of the ICD. Structures of pLGICs solved by x-ray crystallography, cryo-EM, solution NMR, and EPR demonstrate that the ICDs of cation-conducting pLGICs consist of a short loop (L1) that connects M3 with a short helical segment (MX), and a large loop (L2) leading into the membrane associated (MA) and M4 helix (Fig. 1; Kracun et al., 2008; Hassaine et al., 2014; Basak et al., 2018b; Bondarenko et al., 2022).

Within the pLGIC superfamily of >40 subunits in humans, the M3–M4 cytoplasmic polypeptide chains or ICDs exhibit significant diversity in length, amino acid composition, and function. Originally, this domain had been considered largely disordered (Hassaine et al., 2014; Morales-Perez et al., 2016; Basak et al., 2018b; Gharpure et al., 2019; Laverty et al., 2019; Noviello et al., 2021). ICDs regulate gating and ionic conductance (Kelley et al., 2003; Shen et al., 2005; Hales et al., 2006), and they mediate channel assembly and trafficking via interactions with intracellular proteins (Yu and Hall, 1994; Jeanclos et al., 2001; Huebsch and Maimone, 2003; Kracun et al., 2008). Of these intracellular proteins, the endoplasmic reticulum-resident resistance to inhibitors of cholinesterase type 3 protein (RIC-3) was found to affect cell surface expression or functional expression of nicotinic acetylcholine (nAChR) and serotonin type 3A receptors (5-HT_3A_R; Miller et al., 1996; Halevi et al., 2002; Halevi et al., 2003; Castillo et al., 2005; Cheng et al., 2005; Lansdell et al., 2005; Williams et al., 2005; Hales et al., 2006; Walstab et al., 2010; Baptista-Hon et al., 2013; Biaggi-Labiosa et al., 2015; Ben-David et al., 2016).

Expression of RIC-3 in *Xenopus leavis* oocytes enhances the whole-cell current amplitudes of human and rat α7 nAChR but reduces the current amplitudes of human α4β2 and α3β4 nAChRs (Halevi et al., 2002; Halevi et al., 2003), and also of 5-HT_3A_R (Halevi et al., 2003; Jansen et al., 2008; Pirayesh et al., 2020). In contrast, in mammalian cells, RIC-3 robustly enhances the cell surface expression of 5-HT_3A_ receptors and α4β2 and α3β4 nAChRs (Cheng et al., 2005; Lansdell et al., 2005; Williams et al., 2005). The direction of the RIC-3 effect additionally depends on its expression level. At low levels, RIC-3 was found to specifically regulate the assembly of α7 nAChR and to upregulate α4 and β2 subunit expression in a dose-dependent manner; however, at a high levels, RIC-3 inhibits assembly and cell-surface delivery of α7 nAChR; and this inhibitory action of RIC-3 is attributed to its self-aggregation that retains α7 in the endoplasmic reticulum (Cheng et al., 2007; Alexander et al., 2010; Dau et al., 2013).

The extracellular (ECD) and transmembrane (TMD) domains of pentameric neurotransmitter-gate ion channels are long-standing therapeutic targets for psychiatric and neurological disorders. However, the highly homologous sequences and resulting conserved structures of ECDs and TMDs lead to interactions of drug candidates with off-target subunits, and consequently contributed to the discontinuation of many late clinical trials by Pfizer, Astra Zeneca, GlaxoSmithKline, Bristol-Mayer Squibb (Hassaine et al., 2014; Morales-Perez et al., 2016; Basak et al., 2018b; Gharpure et al., 2019; Noviello et al., 2021; Papke and Horenstein, 2021).

To search for an effective subunit-specific approach to regulate the diverse functional properties of pLGICs, we have explored the interaction of the ICD of the 5-HT_3A_ receptor and RIC-3. Our previous studies on this interaction indicate a direct interaction between the ICD and RIC-3 that is mediated via the L1-MX segment of the ICD (Jansen et al., 2008; Goyal et al., 2011; Mnatsakanyan et al., 2015; Nishtala et al., 2016; Pandhare et al., 2016; Pirayesh et al., 2020). However, insights into the interaction sites or the molecular binding motif between RIC-3 and 5-HT_3A_ receptors remain elusive. A refined structural understanding of this interaction is expected to provide the basis for the structure-based design of new chemical entities (NCEs) interfering with this interaction and thus for the development of therapeutics for neurological and psychiatric disorders. Protein–protein interactions have been successfully employed in drug development (Dorr et al., 2005; Antonia et al., 2017; Li et al., 2019; Lu et al., 2020).

With the present study, using alanine-scanning mutagenesis, a protein–protein pull-down assay, and two-electrode voltage-clamp (TEVC) recordings, we provide evidence for the interaction between the 5-HT_3A_ L1-MX segment, as compared to the MBP-5-HT_3A_ L1-MX segment previously used (Nishtala et al., 2016; Pandhare et al., 2016; Pirayesh et al., 2020), and RIC-3 protein. We further demonstrate that further truncation to yield the MX-peptide is sufficient for the interaction with RIC-3. We identify a duplicated motif, DWLR…VLDR, present in both the MX-helix and at the transition of MA and M4-helices, essential for the interaction with RIC-3. Triple Ala substitution, W347, R349, and L353 in MX and W447, R449, and L454 in MAM4 disrupt RIC-3 interaction. In the 5-HT_3A_ structures, these motives are in close proximity at the interface between the lipid bilayer and cytosol.

## Materials and methods

### Mutagenesis

Single amino acid substitutions in the mouse 5-HT_3A_ subunit, W347A, R349A, and L353A and two triple substitutions W347A, R349A, and L353A (or WRLAAA) and W447A, R449A, and L454A (or MA4-WRLAAA) were generated using appropriate primers and the QuikChange II XL Site-Directed Mutagenesis kit (Agilent Technologies) and were confirmed by DNA sequencing. The forward primers for the individual substitutions W347A, R349A, and L353A were 5′-CGG​CCA​GTA​CCT​GACGCACTG​AGG​CAC​CTG​GTC-3′, 5′-CAG​TAC​CTG​ACT​GGC​TGGCACAC​CTG​GTC​CTA​GAC​AG-3′, and 5′-CTG​AGG​CAC​CTG​GTCGCAGAC​AGA​ATA​GCC​TGG-3′, respectively. The forward primer for the triple substitution WRLAAA was 5′-CAG​TAC​CTG​ACGCACTGGCACAC​CTG​GTCGCAGAC​AGA​ATA​GCC​TGG​A-3’. The forward primer for the triple substitution MA4-WRLAAA was 5′-GGCAGTG​GGA​TAC​GTGGCAGAC​AGG​CTG​CTGCTCC-3′. All DNA sequences were confirmed by Genewiz/Azenta. Underlined letters indicate changes in DNA nucleotides/genetic codons, which result in the changes of amino acids of interest at the protein level.

### Complementary RNA (cRNA) production and oocyte injection

Expression plasmid vectors, pGEMHE19 and pXOON, carrying the human RIC3 sequence (GenBank accession no. NM_001206671.2) and mouse 5-HT_3A_ sequence (GenBank accession no. AY605711.1) with V5 epitope tag (GKPIPNPLLGLDSTQ) were generously provided by Dr. Millet Treinin (Hebrew University, Jerusalem, Israel) and Dr. Myles Akabas (Albert Einstein College of Medicine, New York, NY, USA), respectively. Expression plasmid vectors containing substitutions of the mouse 5-HT_3A_ sequence were generated as described in the previous section.

The plasmid vector, 30 μg each, was linearized for 18 h at 37°C in a 250 μl reaction containing restriction enzyme *Nhe*I and CutSmart buffer (New England Biolabs). The linear DNA plasmid was next used to produce cRNA of RIC-3 and 5-HT_3A_ wild type (WT) and engineered receptors. This in vitro transcription process was performed by employing the mMESSAGE mMACHINE T7 Kit (Ambion by Life Technologies). The cRNA was then purified using the MEGAclear kit (Ambion by Life Technologies), dissolved in nuclease-free water, and stored at −80°C until use.

### Expression of proteins in *Xenopus* oocytes

Defolliculated *Xenopus* oocytes were purchased from Ecocyte Bioscience US LLC. Oocytes were transferred to standard oocyte saline (SOS) medium (100 mM NaCl, 2 mM KCl, 1 mM MgCl_2_, 1.8 mM CaCl_2_, and 5 mM HEPES; pH 7.5) supplemented with 1× antibiotic-antimycotic (Cat. #15240-062; Thermo Fisher Scientific/Gibco) and 5% horse serum (Cat. #26050088; Sigma-Aldrich). Oocytes were injected on the same day of the delivery. To express RIC-3, ∼10 ng of cRNA (50.6 μl at 200 ng/μl cRNA) or 50.6 μl of sterile water as control were injected into each oocyte; the oocytes were then incubated at 15°C for 65–68 h before being harvested for plasma membrane isolation. To co-express WT or engineered (“mutant,” MT) 5-HT_3A_ receptors, with RIC-3, 6 ng of 5-HT_3A_-WT or 5-HT_3A_-MT cRNA and 1.5 ng of RIC-3 cRNA were injected into each oocyte, respectively. Oocytes injected with 5-HT_3A_-WT or 5-HT_3A_-MT cRNA alone were used as controls. After the injection, the oocytes were incubated at 15°C for 3 d before they were used for TEVC recordings. SOS medium was replaced 12 h after the injection and subsequently every 24 h.

### Isolation and solubilization of oocyte membranes containing RIC3

To isolate oocyte plasma membranes, oocytes were homogenized using a glass Teflon homogenizer and lysis buffer (25 mM MOPS, 1 mM EDTA, and 0.02% NaN_3_; pH 7.5) supplemented with protease inhibitor cocktail III (Research Products International), 10 μl protease per 1 ml lysis buffer, at a ratio of 20 μl lysis buffer/oocyte. The homogenization was performed by hand until no particles (dark granules) were visible. The homogenized oocytes were then centrifuge at 1,000× *g*, 4°C for 10 min to remove cell debris. Next, the supernatant was transferred to a clean centrifuge tube, while the pellet was resuspended with another 20 μl lysis buffer per oocyte. And the centrifugation was repeated. The second supernatant was combined with the first one, and subsequently, this mixture was spun at 200,000× *g*, 4°C for 45 min to obtain oocyte membranes. After the ultracentrifugation, the membrane pellet was resuspended and washed with lysis buffer containing 1 M NaCl at a ratio of 20 μl buffer per oocyte. This mixture was then spun at 200,000× *g*, 4°C for 45 min to collect the membrane pellet. The resulting pellet was further resuspended and washed in lysis buffer containing 1 M NaCl and 2 mM MgCl_2_ at a ratio of 20 μl buffer per oocyte and pelleted down again at 200,000× *g*, 4°C for 45 min. Afterwards, membrane proteins including RIC-3 were solubilized in solubilization buffer (1.5% Triton X-100, 100 mM K-acetate, 40 mM KCl, 1 mM EDTA, 10 mM MOPS, 0.02% NaN_3_, and 2 mM NEM; pH 7.5) supplemented with the protease inhibitor cocktail III, 10 μl protease per 1 ml lysis buffer, at a ratio of 10 μl buffer per oocyte. The solubilization was carried out for 2 h at 4°C with gently mixing by inverting. After the solubilization was complete, the mixture was centrifuged at 30,000× *g*, 4°C for 1 h. The supernatant was collected, quickly frozen in liquid nitrogen, and stored at −80°C until use.

### Intracellular domain peptides of 5-HT_3_ receptors

**Figure 2.**
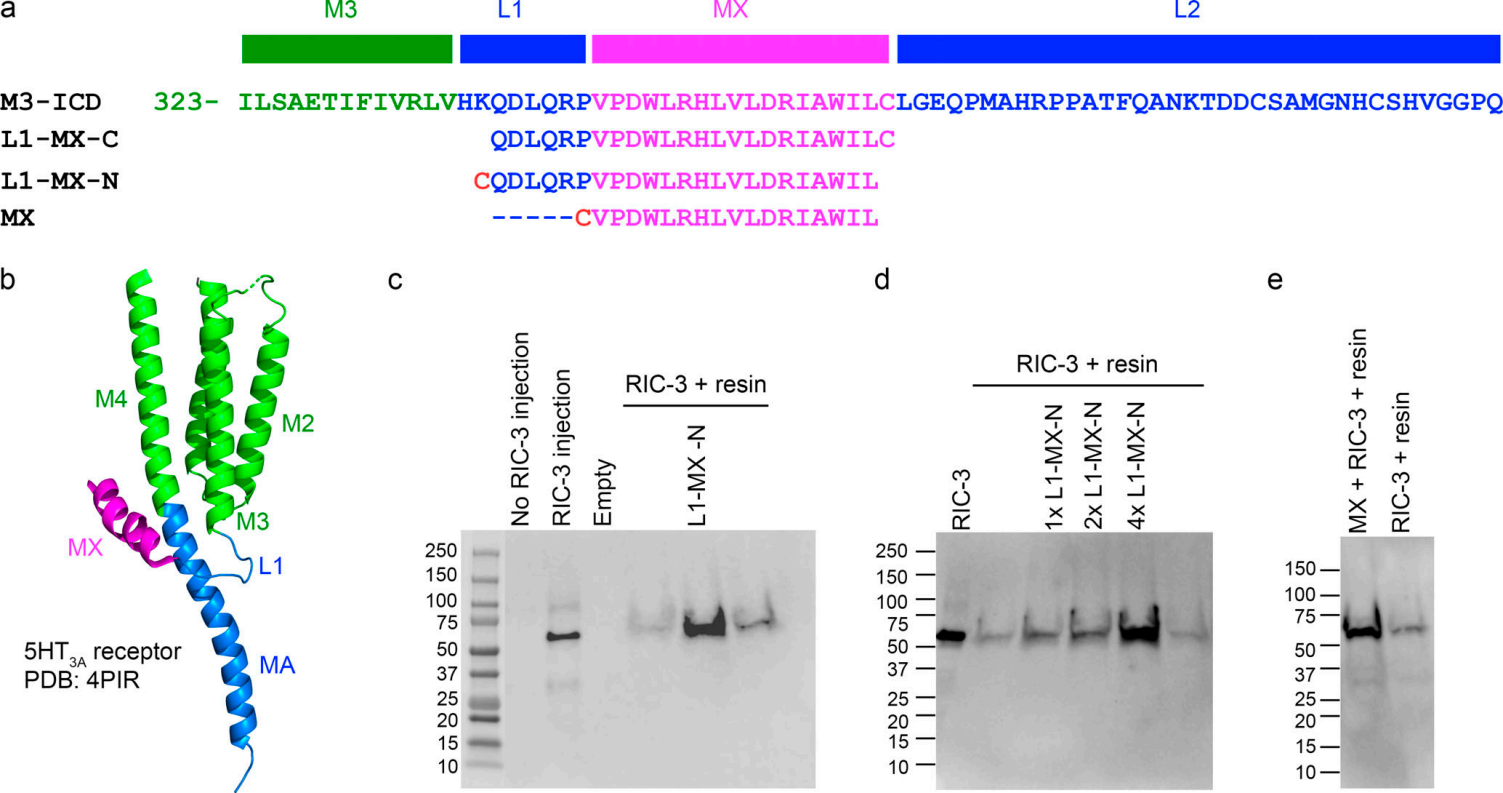
**MX segment binds RIC-3. (a)** Sequence alignment of L1-MX– and MX-peptides with a partial 5-HT_3A_ sequence (top) consisting of the last 13 amino acids of M3 (green), L1-segment (blue), MX-helix (magenta) and 36 residues of the long L2-loop (blue) that connects with the MA-helix (blue, not shown in a, only in b). L1-MX-C contains a C-terminal Cys, and L1-MX-N and MX contain an N-terminal Cys. **(b)** Ribbon representation of a single 5-HT_3A_ subunit indicating L1 and MX and other named peptide segments within the transmembrane and intracellular domains (PDB ID 4PIR; Hassaine et al., 2014). **(c and d)** Eluates of resin-linked peptide pull-downs of RIC-3. Western blot images show RIC-3 expressed in oocytes and control (no RIC-3 injection). Eluates from resin alone show the presence of a minor amount of RIC-3, whereas eluates from resin covalently linked with L1-MX-N peptide show prominent RIC-3 bands. 1×, 2×, and 4× indicate increasing peptide to resin ratios. Eluates from four independent pull-down experiments are presented in lane #6 of c, L1-MX-N, and in lanes #3, 4, and 5 of d, 1× L1-MX-N, 2× L1-MX-N, and 4× L1-MX-N; data shown here are technical replicates that used the same batch of L1-MX-N peptide and RIC-3 extracted from the same batch of *n* = 84 *Xenopus* oocytes. For biological replicates of L1-MX and RIC-3 assay, *n* ≥ 5 were obtained, which used RIC-3 from five different batches of *Xenopus* oocytes and three different batches of L1-MX peptides purchased from two different companies. **(e)** Eluate from MX-linked resin yields RIC-3 band; data shown here is the representative of *n* = 3 biological replicates, where RIC-3 was extracted from three different batches of *Xenopus* oocytes. Source data are available for this figure: SourceData F2.

L1-MX and MX peptides of the ICD from 5-HT_3_ receptors, Fig. 2 A, were designed to contain a C-terminal or N-terminal cysteine (Cys) with a free sulfhydryl (−SH) group. The peptides were synthesized by GenScript or Biomatik and dissolved at a concentration of 2 mg/ml in ice-cold coupling buffer (50 mM Tris and 5 mM EDTA-Na) or DMSO. Tris (2-carboxyethyl) phosphine (TCEP) was added to the solubilized peptides at a final concentration of 2–2.5 mM to prevent oxidation of sulfhydryl groups and maintain sulfhydryls. In case of L1-MX L353A and MX peptides, ice-cold coupling buffer at pH 8.5 and pH 9.5, respectively, was used for initial dissolution; the pH of the peptide solutions was then adjusted to 8.35 and 9.02, respectively, to completely dissolve peptides.

Each peptide concentration was then determined using a nanodrop device (Nanodrop One^C^, Thermo Fisher Scientific) by measuring the absorbance at a wavelength of 280 or 205 nm (Anthis and Clore, 2013), and converted into the respective peptide concentration using the peptide molecular weight, and peptide extinction coefficient using the following equation:

Peptide concentration (mg/ml) = (absorbance/extinction coefficient) × molecular weight.

### Pull-down assay of L1-MX peptides and RIC-3

#### Coupling of L1-MX peptides with iodoacetyl resin

30 μl of settled UltraLink iodoacetyl resin (#53155; Thermo Fisher Scientific) were added to a Pierce spin column (#69705; Thermo Fisher Scientific) and allowed to settle for 5 min at room temperature (RT). The liquid was drained by spinning down at 1,000× *g* for 10 s. The resin was then washed three times with 300 μl of coupling buffer (50 mM Tris and 5 mM EDTA-Na, pH 8.5). After removing the buffer of the third wash, peptide with a N- or C-terminal Cys was added to 30 μl settled resin so that a ratio of resin-bound peptide/resin was between 1.72 mg/ml and 34 mg/ml. This specific ratio was determined and optimized for each peptide. To determine the resin-bound peptide/resin ratio, L1-MX peptides were allowed to react with iodoacetyl resin to form thioether bond between the sulfhydryl group of the peptide and the iodacetyl chains of the resin. The coupling reaction was conducted at RT for 2–5 h depending on each peptide with gently inverting the spin columns for a few seconds every 30 min. Buffer containing unlinked peptide was removed from the spin column by spinning at 1,000× *g* for 10 s. The protein concentration in the flow-through was then determined with the NanoDrop One using the absorbance at 280 or 205 nm (Anthis and Clore, 2013), and peptide concentrations determined as described above. The unlinked peptide mass was calculated from the peptide concentration and the flow through volume. This mass represents unbound peptide and was subtracted with the peptide mass added to the spin column before the coupling reaction to obtain the resin-bound peptide mass. This mass was then divided by 30 μl resin to obtain the ratio of resin-bound peptide/resin.

After the coupling, resin-bound peptides were washed with 300 μl of the coupling buffer, pH 8.5, three times, and available free iodoacetyl sites of the resin were blocked using 120 μl of freshly made 50 mM L-Cysteine containing 25 mM TCEP, pH 8.5. For control pull-down samples, resin samples without coupled peptide were prepared similarly by modifying the iodoacetyl sidechains with Cys. The blocking reaction was conducted at RT with gently inverting spin columns for a few seconds every 10 min for 2 h. The liquid was then removed by spinning at 1,000× *g* for 10 s, and the resin-bound peptide was washed separately with 600 μl of the coupling buffer, 600 μl of 1 M NaCl, and 600 μl of storage PBS buffer (0.1 M Na-phosphate, 0.15 M NaCl, and 0.05% NaN_3_, pH 7.2). The peptide was then stored in the PBS buffer at 4°C until use.

### Pull-down assay with RIC-3

Resin-bound peptides were prepared as described above with a minimum ratio of peptide/resin of 1.72 mg/ml. Storage PBS buffer was removed from the peptides by spinning at 1,000× *g* for 10 s. The resin-bound peptides were then washed three times with 300 μl of binding buffer (0.5% Triton X-100, 100 mM K-acetate, 40 mM KCl, 1 mM EDTA, 10 mM MOPS, and 0.02% NaN_3_; pH 7.5). The binding buffer was removed by spinning at 1,000× *g* for 10 s.

RIC-3 solubilized in 1.5% Triton X-100 was prepared as described above. The protein was thawed on ice for 20 min and then centrifuged at 30,000× *g* for 1 h at 4°C before incubating with the peptide-resins. The supernatant was collected, and 40 μl of this sample was added to 30 μl of either resin alone or peptide-bound resin. The mixture was incubated at 4°C for 2 h. Afterward, 150 μl of washing buffer 1 (1.2% Triton X-100, 100 mM K-acetate, 40 mM KCl, 1 mM EDTA, 10 mM MOPS, and 0.02% NaN3; pH 7.5) was slowly applied to the wall of the spin column without disturbing the resin. The buffer was left in the column for 5 min before being removed at 1,000× *g* for 10 s at RT. In the next step, 60 μl of washing buffer 2 (1 M NaCl, 25 mM Tris-Cl, and 1 mM EDTA; pH 7.5) was slowly applied to the wall of the spin column without disturbing the resin. The buffer 2 was drained by spinning at 1,000× *g* for 10 s at RT.

After washing, RIC-3 protein was eluted from resin-bound peptides by applying 40 μl of elution buffer (277.8 mM Tris-HCl, pH 6.8, 44.4% glycerol, 10% SDS, 10% 2-mercaptoethanol, and 0.02% Bromophenol Blue) and incubating at 75°C for 5 min, before spinning down at 8,000× *g* for 3 min at RT. The flow through was analyzed using western blot/immunoblot as described below to detect the absence or presence of RIC-3.

### Immunoblot analysis

The entire volume of each flow through obtained from the pull-down assay was separated on Mini-PROTEAN TGX gels (Bio-Rad) and proteins transferred to polyvinylidene fluoride (PVDF) membranes (Bio-Rad) using the Bio-Rad trans-blot turbo system following the manufacturer’s protocol. PVDF membranes were briefly immersed in methanol and then equilibrated in western transfer buffer (20% ethanol, 20% of Bio-Rad 5× transfer buffer, #10026938) for 3–5 min. Proteins were then transferred to PVDF membranes at 2.5 amperes and up 25 volts for 4 min at RT. PVDF membranes were blocked under agitation in 5% nonfat milk in tween-containing tris-buffered saline buffer (TTBS buffer: 0.1% Tween-20, 100 mM Tris, and 0.9% NaCl, pH 7.5) for 1 h. Afterwards, the blot was incubated with RIC-3 primary/mouse antibody (Cat. #H00079608-B01P; Abnova Corporation) in a dilution of 1:2,000 overnight at 4°C with gentle shaking. Each blot was then washed three times, each for 5 min, in TTBS before incubation with goat raised anti-mouse secondary antibody, conjugated to horseradish peroxidase enzyme, HRP (#31430; Thermo Fisher Scientific product), in a dilution of 1:5,000 for 2 h, under gentle agitation. Subsequently, the blot was washed five times, each for 5 min, with 20 ml of TTBS buffer. After an additional wash with tris-buffered saline (100 mM Tris and 0.9% NaCl, pH 7.5), SuperSignal West Femto Maximum Sensitivity Substrate (Thermo Fisher Scientific) was used for visualizing RIC-3 signal with a digital imaging system (ChemiDoc MP, Bio-Rad).

### Electrophysiology—*Xenopus* oocyte interaction assay

Changes in the functional surface expression of 5-HT_3A_-WT receptor and Ala substitution constructs in the presence and absence of RIC-3 protein were probed using TEVC recordings 3 d postinjection of oocytes. TEVC data were acquired using a TEV-200A Voltage Clamp amplifier (Dagan Corporation), Digidata 1440A digitizer (Molecular Devices), and Clampex 10.7 with gap-free acquisition mode and 200 Hz sampling rate. Resistances of recording electrodes were in the range of 1–5 MΩ. The ground electrode was made of 1% agarose in 3 M KCl and connected to a bath and a 200 μl perfusion chamber containing Ringer’s buffer or the OR2 buffer (82.5 mM NaCl, 1 mM MgCl, 20 mM KCl, and 5 mM HEPES; pH 7.5). The voltage and current electrodes were filled with 3 M KCl solution. Oocytes were voltage-clamped at a holding potential of −60 mV and continuously superfused under gravity application at 2–5 ml/min. Currents of 5-HT_3A_ receptors and engineered constructs expressed at the cell surface were evoked by applying 10 μM serotonin creatinine sulfate complex (Sigma-Aldrich), or in short 5-HT, in OR2 buffer. Current amplitudes were obtained by subtracting the maximum current in the presence of 5-HT from the baseline or resting current in the absence of 5-HT. Currents recorded from oocytes injected with cRNA for both RIC-3 and 5-HT_3A_ were recorded and analyzed similarly using Clampfit 10.7. Data were analyzed using GraphPad Prism 6 software and the statistical significance was determined using one-way ANOVA and Tukey’s post-hoc test.

## Results

### The 5HT_3A_-MX helix alone interacts with RIC-3

In previous studies, we have demonstrated that RIC-3 directly interacts with the 5HT_3A_ receptor, specifically its ICD, and that the interaction is mediated via the L1-MX segment of the ICD (Nishtala et al., 2016; Pandhare et al., 2016; Pirayesh et al., 2020). In these prior studies, we used recombinant proteins fused with maltose-binding protein (MBP) to facilitate purification and increase stability of the isolated ICDs in the absence of the other domains as fusion partners. Both 6xHIS-MBP-RIC-3 and MBP-5-HT_3A_-ICD constructs in which segments of the ICD were sequentially deleted were expressed individually in *Escherichia coli* and purified to homogeneity to explore protein–protein interaction. We demonstrated that the MBP-5-HT_3A_-L1-MX construct was the smallest construct investigated to mediate RIC-3 interaction. Here, we aimed at investigating the interaction of the 24-amino acid long 5-HT_3A_-L1-MX segment alone, without the highly soluble and solubility enhancing MBP. To establish this modified system, we employed synthetic ICD peptides (Fig. 1 a; and Fig. 2, a and b) covalently linked to iodoacetyl resin and RIC-3 protein heterologously expressed in *Xenopus* oocytes. The initial ICD peptides used were 5-HT_3A_-ICD-L1-MX with either a N- or C-terminal Cys. The Cys sulfhydryl was reacted with the iodoacetyl resin to form a stable thioether linkage. The peptide-resin was then used as the immobile phase together with solubilized RIC-3 protein in the mobile phase in a pull-down type assay. Non-covalently bound proteins were eluted with SDS-containing buffer, separated on SDS-PAGE, and detected with RIC-3 antibody as described in detail in the Materials and method section. Representative data of RIC-3 expression in solubilized oocyte membranes and RIC-3 observed in eluates after pull-down using resin without and with L1-MX peptide covalently bound are shown in Fig. 2 c.

Without the injection of RIC-3 cRNA, no protein band was recognized by RIC-3 antibody in solubilized oocyte membrane samples (Fig. 2 c). In contrast, a strong intensity band migrating just above 50 kD was detected in the lane containing a sample from solubilized membranes of oocytes injected with RIC-3 cRNA. This result strongly indicates the presence of RIC-3 protein in solubilized oocyte membranes. Total membrane samples containing RIC-3 from three oocytes were then used for each pull-down assay reaction (Fig. 2 c, rightmost lanes). Samples using resin reacted with Cys but not L1-MX peptide yielded a faint band indicative of weak/unspecific binding of RIC-3 to resin itself. In contrast, resin modified with L1-MX peptide at a peptide/resin ratio of 7.12 mg/ml, showed a dramatically increased band intensity. The result was consistently observed in at least 10 independent pull-down experiments, and the data from 4 experiments are shown in Fig. 2, c and d. Of note, increasing band intensities were observed with increasing peptide to resin ratios (Fig. 2 d). We used peptide to resin ratios above 4 mg/ml for subsequent experiments when utilizing 30 μl of settled resin to examine the interaction between RIC-3 and L1-MX peptides.

Aiming to define the interaction interface between RIC-3 and 5HT_3A_ receptor, we then shortened the L1-MX peptide by removing the six amino acid long L1-segment (Fig. 2 a). When using resin linked to the 17 amino acid MX-segment via an added N-terminal Cys for the RIC-3 pull-down, we obtained a much stronger band for RIC-3 as compared to the control sample with unlinked resin (Fig. 2 e). We infer that the L1 loop does not play a critical role in the interaction between RIC-3 and the 5HT_3A_-ICD. The MX segment alone fully supports the RIC-3 and 5HT_3A_-ICD interaction.

### Ala substitutions at and between conserved sites, W347, R349, or L353, within the MX helix prevent interaction with RIC-3

**Figure 3.**
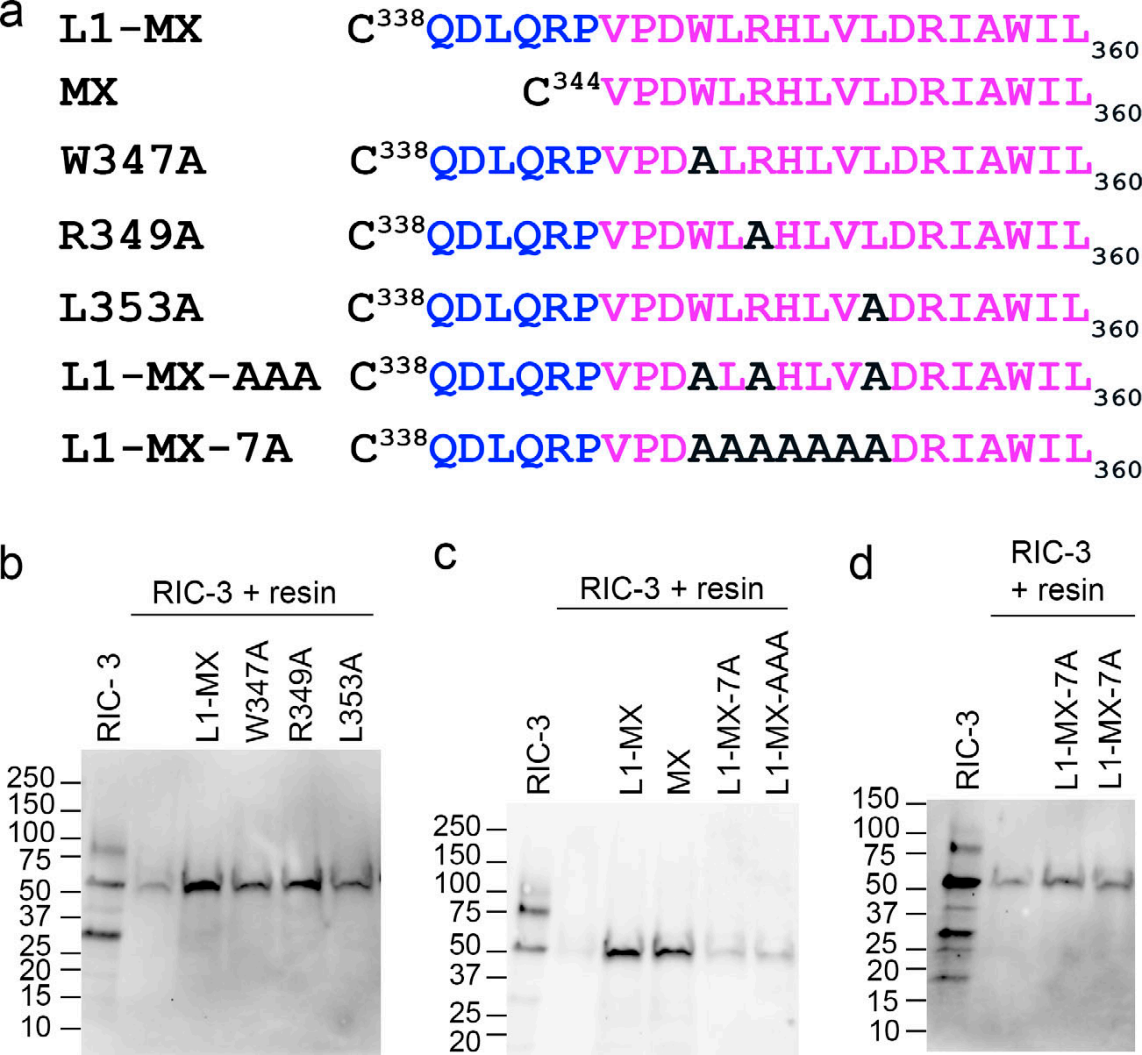
**Ala substitutions at conserved residues disrupt RIC-3 interaction. (a)** Sequence of L1-MX and MX peptides, and single and multiple site substitution peptides W347A, R349A, L353A, L1-MX-AAA, and L1-MX-7A. **(b)** Single-site substitution peptides W347A, R349A, or L353A in the MX helix preserve interaction with RIC-3, albeit with potentially altered affinity based on band intensities. **(c and d)** Multiple Ala substitutions in L1-MX-AAA and L1-MX-7A (two technical replicates) disrupt the interaction with RIC-3. RIC-3 band intensities are comparable to Cys-capped resin background. Data shows representative replicates of *n* ≥ 2 biological replicates and *n* ≥ 3 technical replicates; for L1-MX-AAA and RIC-3 assay, RIC-3 was extracted from a batch of 159 oocytes with *n* = 4 technical replicates. Source data are available for this figure: SourceData F3.

To characterize the RIC-3 binding interface within the 5-HT_3A_ MX segment, we used alanine scanning to generate single and multiple Ala replacements at conserved positions within the MX segment (Fig. 3 a). Residues Trp W347, Arg R349, and Leu L353 in MX are conserved across species and subunits of cation-conducting pentameric ligand-gated ion channels. These Ala substituted peptides were immobilized on iodoacetyl resin as described above and used to probe RIC-3 binding. Single Ala substitutions W347A, R349A, or L353A in the MX segments gave rise to RIC-3 bands with a signal much stronger than that of the unspecific binding between resin and RIC-3. Under these conditions, the single Ala substitutions have little to no effect on the interaction between L1-MX and RIC-3 (Fig. 3 b). However, the combined substitution of Ala residues for either seven amino acids from W347 to L353 (MX-7A peptide, Fig. 3 a) or three conserved positions W347, R349, and L353 (MX-AAA peptide, Fig. 3 a) in the MX segment drastically reduced the intensity of the RIC-3 bands to that of the unspecific or background binding between unmodified resin and RIC-3 (*n* = 3). We infer that MX-7A and MX-AAA substitutions abolish the interaction with RIC-3 (Fig. 3, c and d). Our data suggest that substitutions of conserved residues, W347, R349, and L353 in the MX segment, together, disrupt the interaction between ICD and RIC-3.

To address how the Ala substitutions at these conserved positions affect the function of the 5-HT_3A_ receptor and the modulation of its functional surface expression by RIC-3, we introduced these substitutions, W347A, R349A, and L353A, in full-length 5-HT_3A_ receptors and examined changes in the receptors’ function using *Xenopus* oocytes and TEVC recordings.

### Impact of Ala substitutions at W347, R349, and L353 on RIC-3 modulation of functional surface expression of 5-HT_3A_ receptors

**Figure 4.**
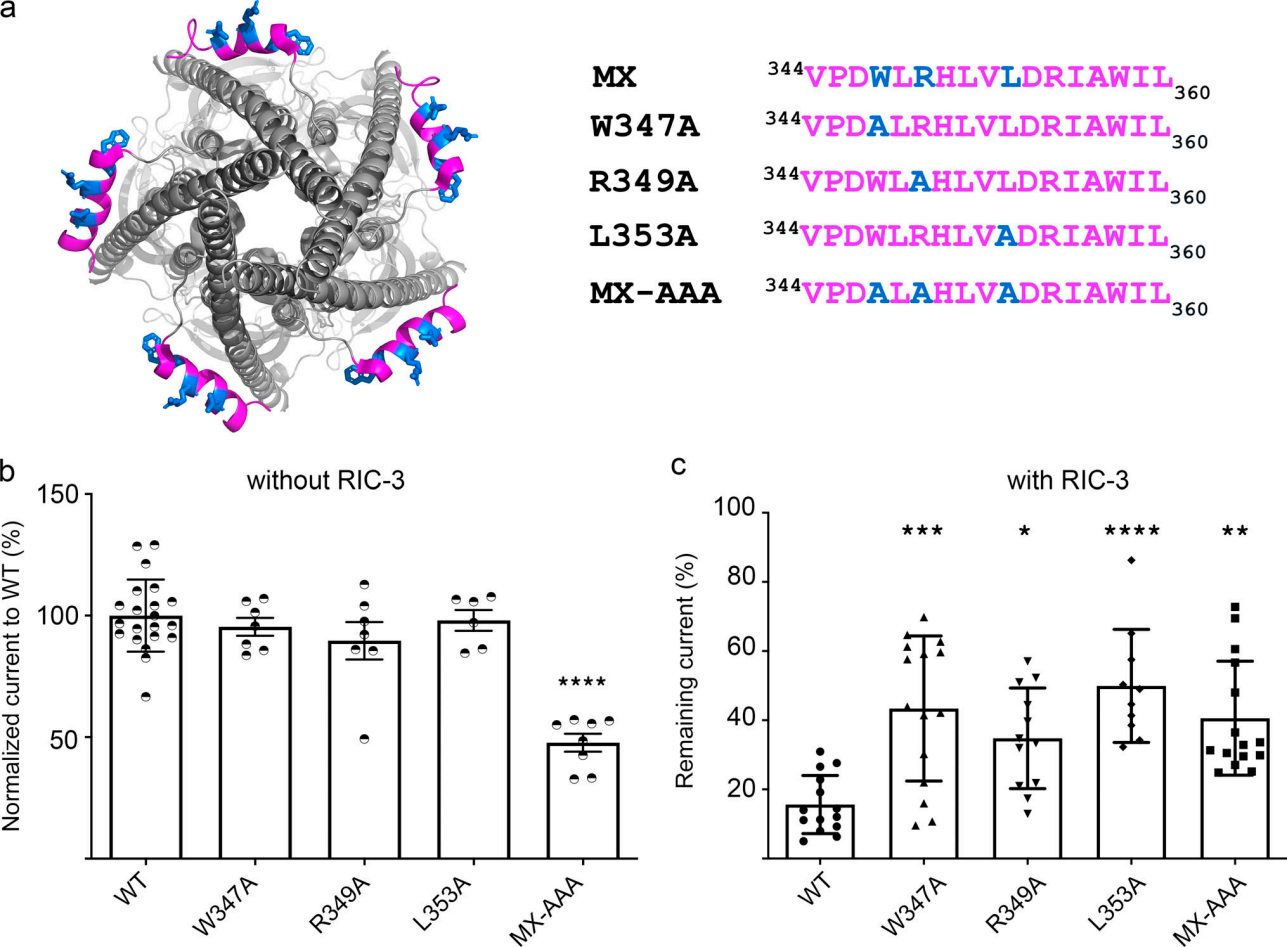
**Ala substitutions within the MX-segment reduce modulation of functional 5-HT**_**3A**_ **surface expression by RIC-3. (a)** View of 5-HT_3A_ channel (PDB ID 6W1J; Basak et al., 2020) from the intracellular side, with residues W347, R349, and L353 in blue (left), and alignment of the MX-segments with Ala substitutions as indicated (right). **(b)** Serotonin-induced current amplitudes of all constructs normalized to the current amplitude of WT 5-HT_3A_ receptor. The triple Ala substitution leads to significantly reduced current amplitudes; biological replicates *n* = 21 for WT, *n* = 7 for W347A, *n* = 7 for R349A, *n* = 6 for L353A, *n* = 8 for MX-AAA. **(c)** Current amplitudes for each construct co-expressed with RIC-3 normalized to the amplitudes of each channel expressed alone, respectively; biological replicates *n* = 14 for WT, *n* = 15 for W347A, *n* = 12 for R349A, *n* = 10 for L353A, *n* = 15 for MX-AAA. Statistical significance in b and c was determined vs. WT using ordinary one-way ANOVA with Tukey’s post-hoc test. Significance is indicated: * P = 0.0485, ** P = 0.001628, *** P = 0.000321, **** P = 0.00005 (c: L353A vs. WT), **** P = 0.000000000018616 (b: MX-AAA vs. WT).

We generated four constructs of 5-HT_3A_ receptors, i.e., three single Ala substitutions (W317A, R349A, or L353A) and one triple substitution (W317A, R349A, and L353A; Fig. 4 a). cRNA for these engineered constructs and the WT 5-HT_3A_ receptor were injected into *Xenopus* oocytes, either alone or with cRNA for RIC-3, at a ratio of 6 to 1.5 ng as examined previously (Pirayesh et al., 2020). 3 d post-injection, the oocytes were used to examine the inhibitory effect of RIC-3 on the functional surface expression of 5-HT_3A_ channels by eliciting whole-cell currents with 10 μM of serotonin (5-HT). At least six oocytes from two to four different batches of oocytes were used for each type of 5-HT_3A_ receptor. Without the injection of RIC-3 cRNA (Fig. 4 b), no significant differences in current amplitudes were observed between the WT channels and 5-HT_3A_ channels with a single Ala substitution, W347A, R349A, or L353A. However, the current amplitude elicited from 5-HT_3A_ channels containing the MX-AAA substitution was reduced to 47.7% ± 0.1 compared to WT (one-way ANOVA with Tukey’s post hoc test, P = 0.000000000018616). Our data suggests that single Ala substitutions W347A, R349A, or L353A do not interfere with the maximum current amplitude of the 5-HT_3A_ channels, whereas the triple substitution W347A, R349A, and L353A significantly reduces current amplitudes compared to WT.

When 5-HT_3A_ channels were co-injected with RIC-3, we observed that RIC-3 inhibits 5-HT_3A_ current amplitudes elicited by serotonin in *Xenopus* oocyte as reported in previous studies (Halevi et al., 2003; Jansen et al., 2008; Pirayesh et al., 2020). The RIC-3 inhibitory effect was calculated as “remaining current” for each pair of 5-HT_3A_ receptors as follows: remaining current (%) = the peak current amplitude of 5-HT_3A_ channels in the presence of RIC-3 divided by the peak current amplitude from 5-HT_3A_ channels alone. This remaining current is used to assess the interaction between RIC-3 and the respective 5-HT_3A_ WT or modified receptor. For WT 5-HT_3A_ channels, co-expression with RIC-3 leads to remaining currents of 15.62% ± 0.08. In contrast, the remaining currents for the single substitutions W347A, R349A, or L353A co-expressed with RIC-3, 43.4% ± 20.98, 33.9% ± 14.8, and 50% ± 16.33, respectively, showed a significantly reduced impact of RIC-3 compared to WT (one-way ANOVA with Tukey’s posttest, P = 0.000321, P = 0.0485, P = 0.00005 (Fig. 4 c). The remaining current of MX-AAA 5-HT_3A_ channels, 40.59% ± 0.16, was 2.6 times higher than that in WT 5-HT_3A_ channels (one-way ANOVA with Tukey’s posttest, P = 0.001628; Fig. 4 c). Significant changes in the inhibitory action of RIC-3, thus, were observed in all constructs containing Ala substitutions in the MX segment compared to WT channels. This result indicates that Ala replacements at residues W347, R349, and L353 weakened the inhibitory effect of RIC-3 on the functional surface expression of 5-HT_3A_ channels.

Interestingly, the MX-AAA substitution’s impact on the interaction observed in the pull-down assay using an isolated 5-HT_3A_ segment and the in vivo assay using full-length 5-HT_3A_ subunits is not equivalent quantitatively: the interaction was abolished in pull-down assay as compared to attenuated in the functional surface expression assay.

**Figure 5.**
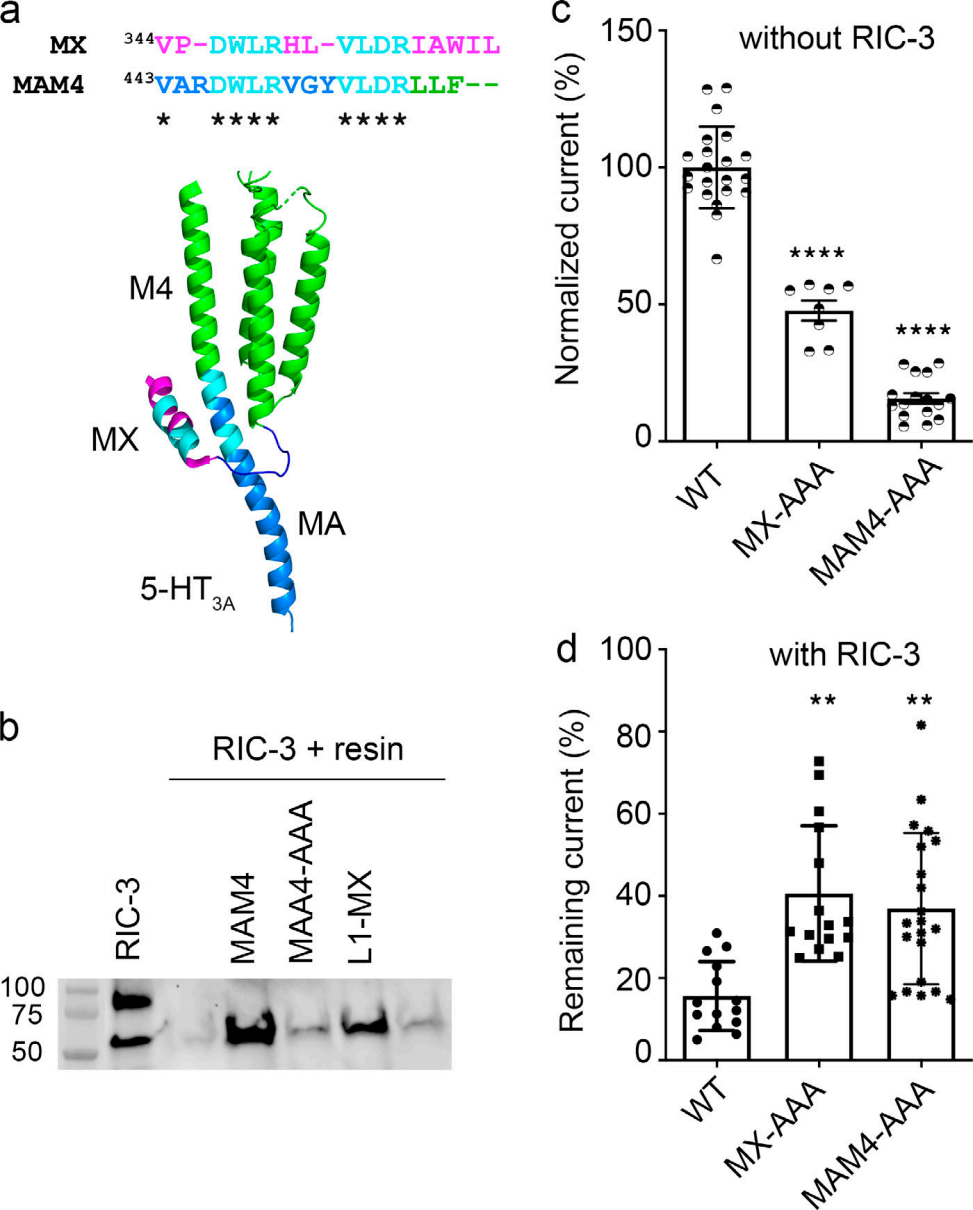
**A duplicated RIC-3 binding motif at the MA–M4 transition mirrors the effects observed for the MX-segment RIC-3 site. (a)** Duplicated RIC-3 binding motif, DWLRxxxVLDR, depicted on the cartoon structure of 5-HT_3A_ transmembrane and intracellular domains: two amino acid clusters (cyan) consisting of Asp, Trp, Leu, Arg (DWLR) and Val, Leu, Asp, Arg (VLDR), respectively, are present in both MX-helix and straddling the MA–M4 helices. **(b)** Triple Ala substitution at residues W447, R449, and L454 of the MAM4 peptide reduces the RIC-3 band intensity to background levels. **(c)** Serotonin-induced current amplitudes for AAA substitutions normalized to WT currents show significantly reduced amplitudes as compared to WT 5-HT_3A_ receptor; biological replicates *n* = 21 for WT, *n* = 8 for MX-AAA, *n* = 16 for MAM4-AAA. **(d)** Remaining 5-HT current for each construct in the presence of RIC-3. AAA substitutions lead to less decreased currents with RIC-3 co-expression as compared to WT; biological replicates *n* = 14 for WT, *n* = 15 for MX-AAA, *n* = 21 for MAM4-AAA. Statistical significance in c and d was determined vs. WT using ordinary one-way ANOVA with Tukey’s post-hoc test. Significance is indicated as **** P = 0.000004 (c: MX-AAA vs. MAM4-AAA), **** P = 0.000000000017930 (c: MAM4-AAA vs. WT), ** P = 0.001628 (d: MX-AAA vs. WT), ** P = 0.005000 (d: MAM4-AAA vs. WT). Source data are available for this figure: SourceData F5.

This difference in interactions of RIC-3 with either L1-MX peptides containing Ala substitutions or full-length 5-HT_3A_ mutants lead us to question if there is more than one binding area for RIC-3 within the 5-HT_3A_ subunit. We identified a duplication of the highly conserved MX-helix WRL motif (D**W**L**R**…V**L**DR) at the transition between MA and M4 helices (Fig. 5 a). In the MA–M4 transition the corresponding residues are W447, R449 and L454. Bold letters indicate residues that were replaced by Ala residues in pull-down assay and TEVC experiments. These residues are also critical for the interaction between 5-HT_3A_ ICD and RIC-3 protein, which is the result of this report.

### Duplicated RIC-3 motif at the MA–M4 transition

To test whether the duplicated motif in the MA–M4 transition presents a second binding site for RIC-3 within the 5-HT_3A_ subunit, we generated peptides and constructs containing Ala substitutions for residues W447, R449, and L454. We then examined the impact of the Ala substitutions on the interaction with RIC-3 and the functional surface expression of the channels (Fig. 5 b). Using L1-MX peptide and peptide-unmodified resin as the controls, we observed a strong RIC-3 signal in the pull-down assay using MAM4 peptide, which was almost abolished for MAM4-AAA. The Ala replacements at residues W447, R449, and L454, thus, disrupted the interaction between RIC-3 and MAM4 peptide similarly to the Ala replacements in L1-MX peptide (Fig. 3 c).

In the absence of RIC-3 co-expression, full-length 5-HT_3A_ channels containing the triple substitution MAM4-AAA produced serotonin-induced currents with the amplitude equivalent to 15.65% ± 0.08 of the currents elicited for WT, which is lower than the currents for the MX-AAA construct, 47.7% ± 0.1 (Fig. 5 c). This data reveals that W447, R449, and L454 residues play a critical role in the functional expression of 5-HT_3A_ channels (WT vs. MAM4-AAA, one-way ANOVA with Tukey’s posttest, P = 0.000000000017930). Residues W447, R449, and L454 also possess a stronger inhibitory effect on the 5-HT_3A_ functional expression than residues W347, R349, and L353 (one-way ANOVA with Tukey’s posttest, P = 0.000004).

When being co-expressed with RIC-3, the full-length 5-HT_3A_ receptors containing W447A, R449A, and L454A in the MA–M4 transition produced a remaining current of 36.88% ± 0.18 (Fig. 5 d). This inhibitory effect of RIC-3 is similar to the one determined for MX-AAA 5-HT3A channels with 40.59% ± 0.16 seen in (Fig. 4 c and Fig. 5 d). Both of these remaining currents for MX-AAA and MAM4-AAA are significantly higher than the ones observed for WT channels, 15.62% ± 0.08, as reported above (Fig. 4 c and Fig. 5 d).

Our findings thus suggest a second location in 5-HT_3A_ ICD, the MA–M4 transition, which interacts with RIC-3 in addition to the MX.

Removing both RIC-3 interaction locations in 5-HT3A would be expected to completely eliminate the effect of RIC-3 co-expression to reduce functional surface expression. The construct with six substituted positions (W347A, R349A, L353A W447A, R449A, and L454A) did not yield serotonin-induced currents either in the absence or presence of RIC-3.

## Discussion

Using pull-down assay, Ala replacement, and TEVC recordings, we here report a binding motif for RIC-3, DWLR…VLDR, which is located at two different locations in the intracellular domain of the mouse 5-HT_3A_ receptor, MX and MA-M4 segments.

Our previous studies using swapped domains/chimeric constructs demonstrated that RIC-3 interacts with 5-HT_3A_ receptors at the intracellular domain to regulate functional surface expression of 5-HT_3A_ channels (Jansen et al., 2008; Goyal et al., 2011; Mnatsakanyan et al., 2015; Nishtala et al., 2016). Subsequently, we used a sequential deletion approach to determine the minimal length of 5-HT_3A_-ICD, the L1-MX fragment, that is critical for the interaction with RIC-3 protein (Pandhare et al., 2016; Pirayesh et al., 2020).

In this work, we report that the MX segment is critical for the interaction with RIC-3 (Fig. 2 e), and when residues W347, R349, and L353 in MX are replaced with Ala, the replacement interrupts the interaction with RIC-3 (Fig. 3 c). When single residue W347, R349, or L353 or triple residues W347, R349, and L353 are replaced by Ala, the replacements attenuate the inhibitory effect of RIC-3 on the functional surface expression (Fig. 4 c). Our findings thus strongly indicate that MX houses the binding site for RIC-3 protein, and residues W347, R349, and L353 take part in the interaction with RIC-3. These residues belong to two amino acid clusters D**W**L**R** and V**L**DR, which are also found in the MA-M4 segment of the mouse 5-HT_3A_ ICD (Fig. 5 a) or the human 5-HT_3A_ ICD (UniProt accession no. P46098). Overall, the full-length 5-HT_3A_ and RIC-3 interaction studies corroborate that W347, R349, and L353 are important for RIC-3 interaction.

Our work also examined the role of residues W447, R449, and L454 in the amino acid clusters D**W**L**R** and V**L**DR in MA-M4 of the mouse 5-HT_3A_ ICD using pull-down assay, Ala replacement, and TEVC recordings. We found similar effects of residues W447, R449, and L454 on the interaction with RIC-3 or on the inhibitory modulation of RIC-3 on functional surface expression of 5-HT_3A_ channels, as seen with residues W347, R349, and L353 in MX. Our findings thus suggest that the D**W**L**R**…V**L**DR motif is critical for the interaction with RIC-3, and this motif is located at two different sites in the ICD. The next question is why this motif exists twice in the ICD.

When examining the role of residues W347, R349, and L353 in the MX segment or W447, R449, and L454 in the MA-M4 segment using TEVC in the absence of RIC-3 co-expression, we notice that the Ala replacement in MX results in significantly larger current amplitude as compared to the construct with the triple Ala substitution in MA–M4 (Fig. 5 a, one-way ANOVA with Tukey’s posttest, P < 0.0001). In other words, residues W447, R449, and L454 possess a stronger inhibitory effect on the 5-HT_3A_ functional expression than residues W347, R349, and L353. While this result can arise from a change in conformation of the channels due to the mutations, the existence of a duplicated motif in the ICD, which interacts with RIC-3, also points to the possibility that RIC-3 interact with MX of the ICD to perform a different function from the interaction of RIC-3 with MA–M4 of the ICD. Our findings related to the impact of the MX segment on functional expression is reminiscent of the work of Hales and co-workers, which suggests that when 10 amino acids in MX of the human 5-HT_3A_ receptor were deleted, either residues PA**W**L**R**HLV**L**E or residues L**R**HLV**L**ERIA, the functional surface expression of human 5-HT_3A_ receptor in HEK cells was abolished (Baptista-Hon et al., 2013).

**Figure 6.**
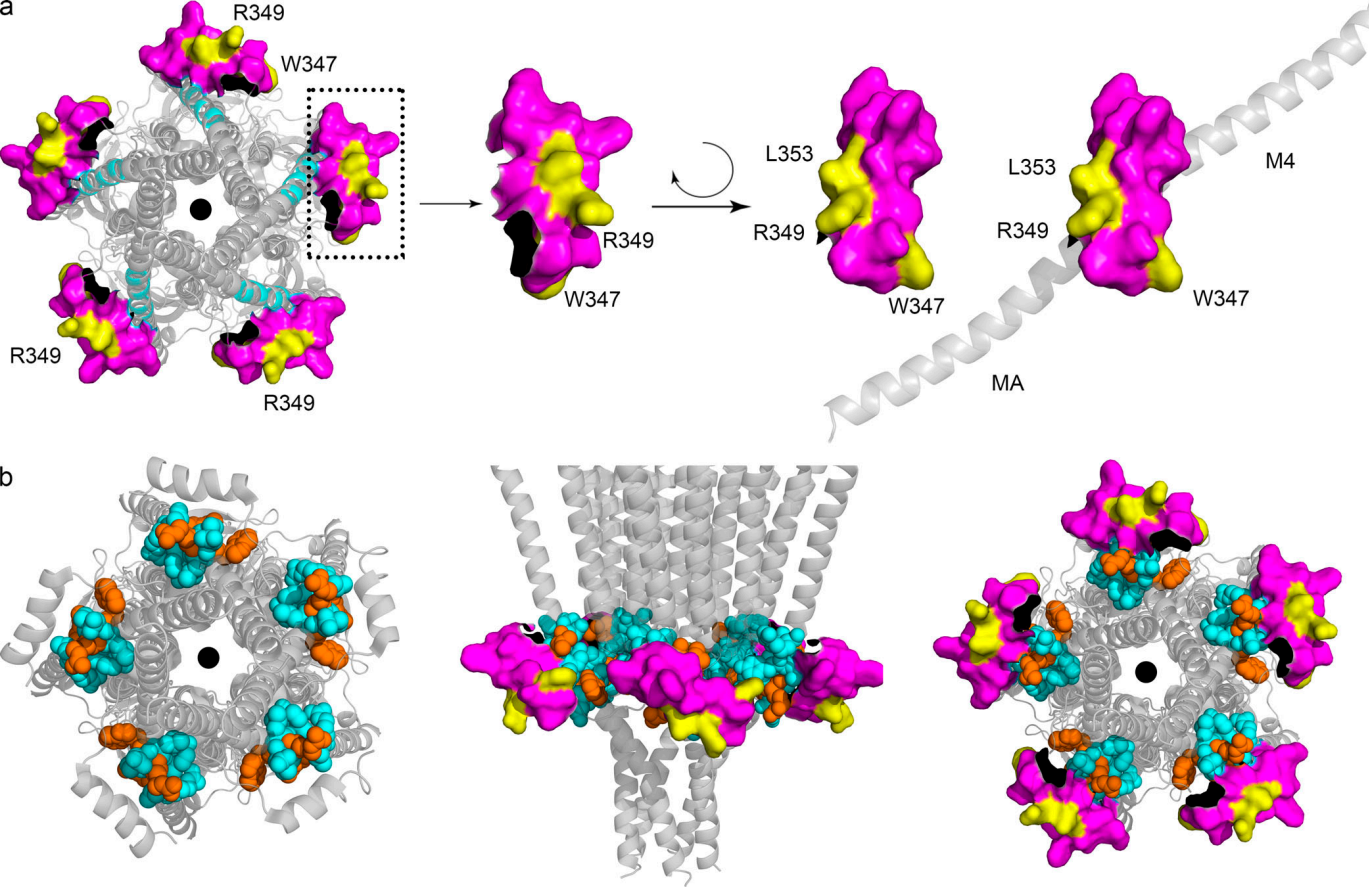
**Visualization of RIC-3 binding motifs in the MX segment and in the MA–M4 transition. (a)** Intracellular view of the pentameric 5-HT_3A_ structure. MX-helix is in magenta space filling representation, and amino acids W347, R349, and L353 of MX are in yellow. **(b)** Intracellular view with the MA–M4 transition in cyan and orange, and residues W447, R449, and L454 of MA-M4 are in orange, left; view parallel to the membrane in addition with MX segment in magenta and yellow as in a, middle; and intracellular view with the same color representation, right (PDB ID 6W1J; Basak et al., 2020).

The relative spatial orientation of both MX and MA–M4 sites is visualized in Fig. 6. Intriguingly, the two duplicated motifs are in close proximity, with the involved helices almost packing against each other but the key sidechains accessible on the surface. The sidechains of the conserved residues Trp, Arg, and Leu (WRL) in MX (Fig. 6 a) point outward to the surrounding environment (close to the headgroup region of the lipid bilayer) in an orientation that primes these residues for participation in an interaction with a 5-HT_3A_ binding partner like RIC-3 that is also a transmembrane protein. This outward orientation of the WRL sidechains is observed in the majority of available structures of drug-bound or resting state 5-HT_3A_ receptors (PDB IDs 4PIR, 6BE1, 6DG7, 6DG8, 6NP0, 6W1J). Residues Trp, Arg, and Leu in MX are conserved across species and subunits of cation-conducting pentameric ligand-gated ion channels. The corresponding residues in MX of nAChR α7 subunits (W330, R332, and L336, UniProt accession no. P36544), also face outward to the surrounding environment (PDB ID 7KOQ). Previous studies demonstrate that RIC-3 enhances the functional expression of nAChR α7 (Castillo et al., 2005; Gu et al., 2016; Kweon et al., 2020). Of note, in many expression hosts there is only negligible nAChR α7 functional surface expression in the absence of endogenous or exogenous RIC-3 expression. nAChR α7 subunits only contain the conserved MX RIC-3 binding motif but not the duplicated motif in MAM4. The opposing effects of RIC-3 co-expression on nAChR α7 (increased expression) and 5-HT3A-MAM4-AAA (decreased expression) when only the MX site is present, indicate that the directionality of the effect depends on factors other than the pLGIC subunit RIC-3 interaction interface itself. Clearly, more studies are needed to dissect the molecular mechanism of RIC-3’s modulation of pLGIC functional surface expression.

On another note, to the best of our knowledge, this is the first time the functional role of residues W347A, R349A, and L353A or residues W447, R449, L454 has been reported. Previous studies on the M3-M4 loop or ICD of 5-HT_3A_ receptors or acetylcholine receptors from other groups did not specifically note a functional significance of W347, R349, and L353 residues (Yu and Hall, 1994; Kelley et al., 2003; Shen et al., 2005; Hales et al., 2006; Kracun et al., 2008; McKinnon et al., 2011; McKinnon et al., 2012; Baptista-Hon et al., 2013).

### Conclusion

Here we identify a sequence motif in 5-HT_3A_ channels within the intracellular domain MX segment that mediates interaction with RIC-3. The motif is conserved across species and only observed in cation-conducting family members of pGLICs. Anion conducting family members do not contain this motif and their functional surface expression is also not modulated by RIC-3 co-expression. Intriguingly, we observe a duplication of this MX-helix binding motif DWLR…VLDR for RIC-3 at the MAM4 transition that is unique for 5-HT_3A_ subunits, but not other subunits. Within the completely folded pentameric 5-HT_3A_ channel, these two motifs are in close proximity. Our findings provide detailed insights into the interaction between the intracellular domain of pGLICs and RIC-3 that can be used to guide drug design aiming at regulating the functional expression of pLGICs and to further guide mechanistic studies of RIC-3 pLGIC modulation.

## Supplementary Material

SourceData F2 is the source file for Fig. 2.

SourceData F3 is the source file for Fig. 3.

SourceData F5 is the source file for Fig. 5.

## Data Availability

Representative data for Figs. 2, 3, and 5 are available in the published article and its online supplemental material. Western blot data for biological or technical replicates, as well as the original TEVC recording files for Figs. 4 and 5 are available from the corresponding author upon reasonable request.

## Data availability

Representative data for Figs. 2, 3, and 5 are available in the published article and its online supplemental material. Western blot data for biological or technical replicates, as well as the original TEVC recording files for Figs. 4 and 5 are available from the corresponding author upon reasonable request.
