# Supplementary figures and images for: Binding motif for RIC-3 chaperon protein in serotonin type 3A receptors
Source: J Gen Physiol. 2023 Apr 7;155(6):e202213305. doi: 10.1085/jgp.202213305 (PMC10083716; doi:10.1085/jgp.202213305)

Fig. 2c

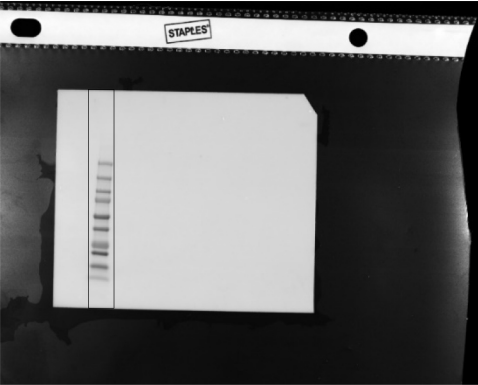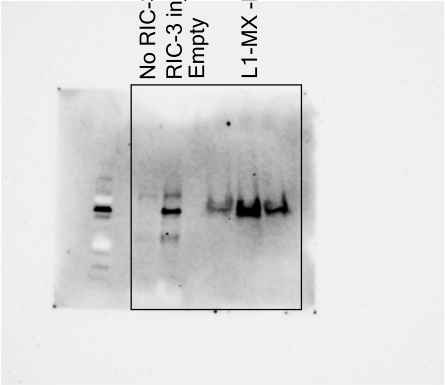

Fig. 2d

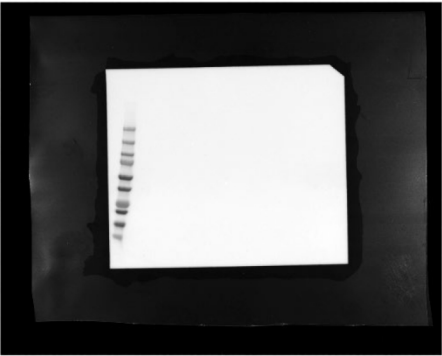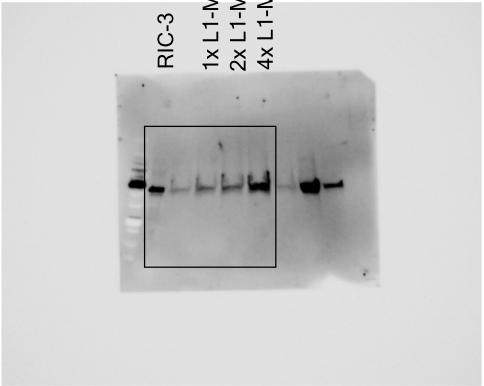

Fig. 2e

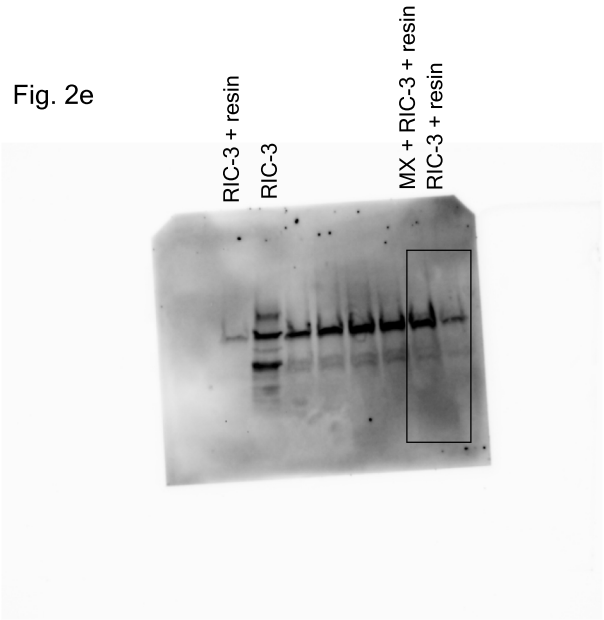

Supplement: SourceData F2 — is the source file for Fig. 2. [file JGP_202213305_SourceDataF2.pdf]

Fig. 3b

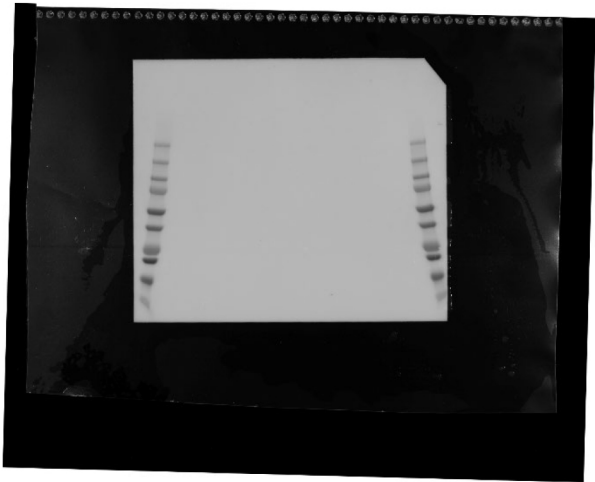

RIC-3 + resin

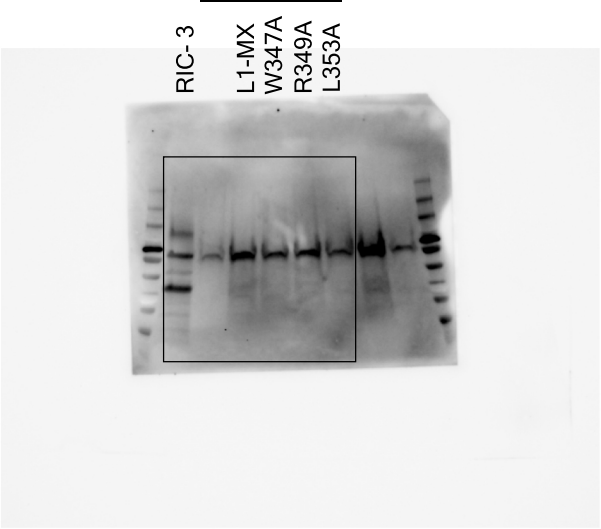

Fig. 3d - ladder

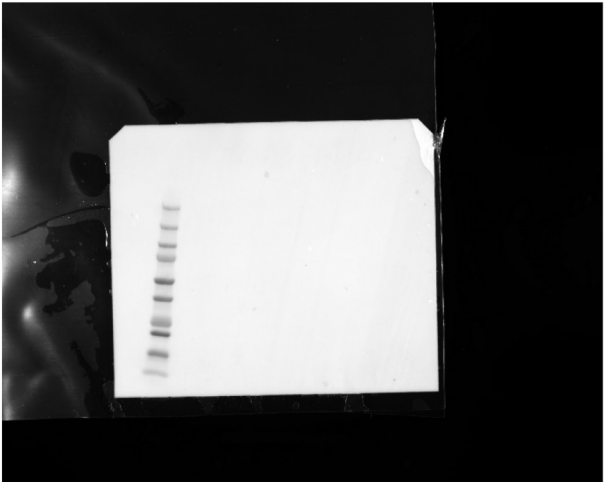

Fig. 3d - blot

Fig. 3c

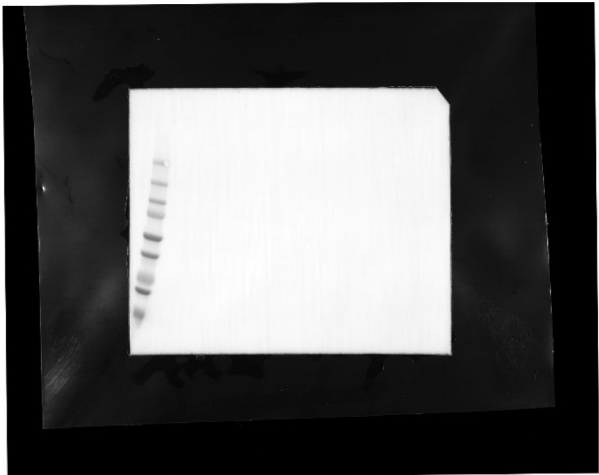

RIC-3 + resin

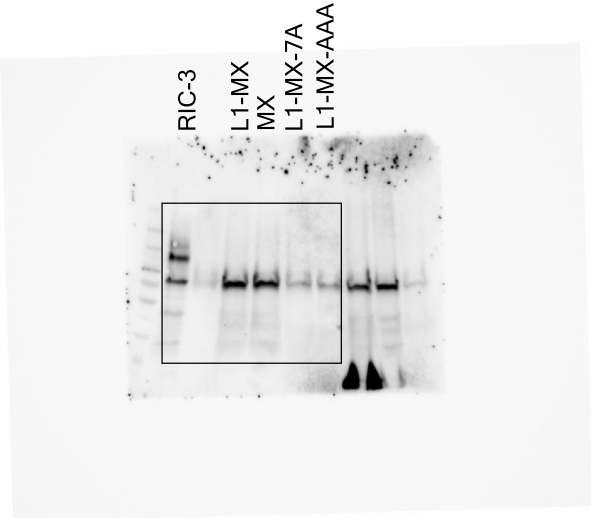

RIC-3  
+ resin

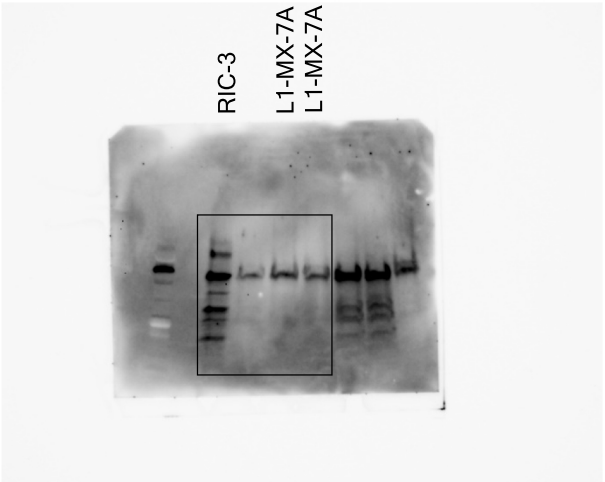

Supplement: SourceData F3 — is the source file for Fig. 3. [file JGP_202213305_SourceDataF3.pdf]

Fig. 5b

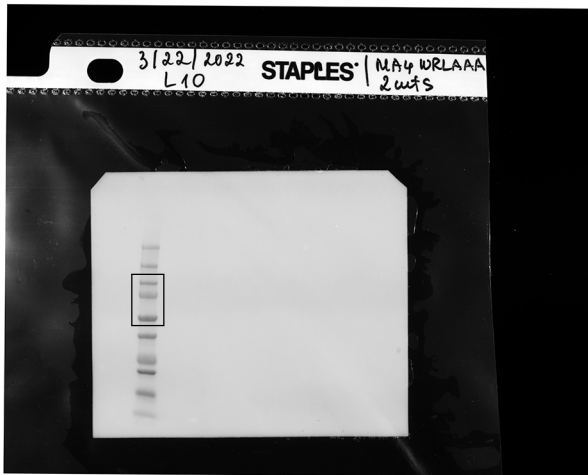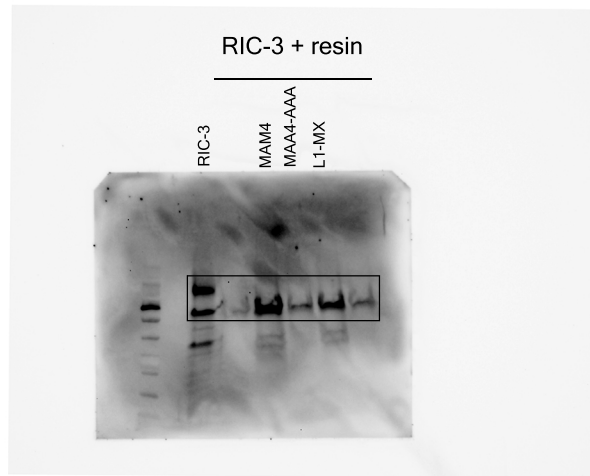

Supplement: SourceData F5 — is the source file for Fig. 5. [file JGP_202213305_SourceDataF5.pdf]
